# 61 A Burn Center’s Experience with COVID-19 Positive Burn Patients

**DOI:** 10.1093/jbcr/irac012.064

**Published:** 2022-03-23

**Authors:** Melissa M McLawhorn, Bonnie C Carney, Sarah E Burkey, Cara Delatore, Melissa Brantley, Lauren T Moffatt, Laura S Johnson, Jeffrey W Shupp

**Affiliations:** MedStar Health Research Institute, Washington, District of Columbia; Medstar Health Research Institute, Washington DC, District of Columbia; MedStar Health Research Institute, Washington, District of Columbia; MedStar Health Research Institute, Washington, District of Columbia; MedStar Washington Hospital Center, Washington, District of Columbia; Burn Center at MedStar Washington Hospital Center, Washington DC, District of Columbia; MedStar Washington Hospital Center / Georgetown School of Medicine, Washington, District of Columbia; MedStar Washington Hospital Center, Washington DC, District of Columbia

## Abstract

**Introduction:**

The emergence of SARS-COV-2 and the COVID-19 pandemic has complicated the presentation, treatment, and prognosis of all types of patients. Further characterization and analysis of how concomitant COVID-19 infection impacts different patient populations is important for improving treatment strategies. Patients with burn injures often require ICU-level care, mechanical ventilation, and extensive surgical intervention. Concomitant COVID-19 infection in this population presents a new challenge to clinical teams. The purpose of this project is to compare COVID-19 positive burn patients treated at a regional burn center with those that are not.

**Methods:**

Following IRB approval, our institution’s burn registry was queried from March 2020-June 2021. Data on demographics, injury circumstances, COVID-19 status, and outcomes were collected. Continuous variables were nonparametric and\compared using Mann-Whitney U test. Categorical variables were compared using Chi-squared with Fischer’s Exact test, where appropriate.

**Results:**

Of the 622 patients admitted at our institution, 19 tested positive for COVID-19 during their hospitalization. Demographic and injury information is reported in Table 1. There were statistically significant differences between the COVID-19 positive and negative groups in regard to race and presence of inhalation injury (p=0.0002, p=0.0002). The TBSA burned was slightly higher in the COVID-19 positive group (9.1 vs 6.7%). COVID-19 positive patients spent more time ventilated (48±32.5 vs.12.2 ± 16.2 days, p=0.0035**) and had both longer ICU (42.71±37.41 vs 11.1±15.4 days, p=0.0175*) and hospital (26.32±32.14 vs 8.177±11.95 days, p< 0.0001***) lengths of stay (LOS). No COVID-19 positive patients died while 5% of the COVID-19 negative patients did. All outcomes were statistically significant.

**Conclusions:**

Despite similar TBSA injury burden and age breakdown, patients at our institution who tested positive for COVID-19 required more time on the ventilator and were hospitalized longer. People of color had a higher percentage of positive tests than their Caucasian counterparts. While mortality rates were higher in the COVID-19 negative cohort, morbidities associated with longer LOS must be considered.

